# A chromosomal analysis of three species of *Timarcha* (Coleoptera, Chrysomelidae, Chrysomelinae)

**DOI:** 10.3897/CompCytogen.v10i1.5570

**Published:** 2016-01-22

**Authors:** Eduard Petitpierre

**Affiliations:** 1 Dept. Biologia, Universitat de les Illes Balears, 07122 Palma de Mallorca, Spain

**Keywords:** Coleoptera, Chrysomelidae, Chrysomelinae, karyotypes, Timarcha, evolution

## Abstract

The karyotypes of three species of *Timarcha* Latreille, 1829 have been analysed. Timarcha (Metallotimarcha) metallica (Laicharting, 1781), has 18 + Xy_p_ male meioformula and 2n = 38 chromosomes, similar to those found in the two species of subgenus *Americanotimarcha* Jolivet, 1948, in agreement with morphological and molecular phylogenetic grounds. Timarcha (Timarcha) carmelenae Petitpierre, 2013 displays 9 + Xy_p_ and 2n = 20 chromosomes as in morphologically related Andalusian species, whereas Timarcha (Timarcha) parvicollis ssp. seidlitzi Kraatz, 1879 shows 11 + Xyp and 2n = 24 chromosomes, clearly differing from the previous species. These results are discussed in order to get an insight into the main trends of the chromosomal evolution in *Timarcha*.

## Introduction

The highly speciose genus *Timarcha* Latreille, 1829 comprises more than three hundred described taxa, almost all from the Palaearctic (Gómez-Zurita 2008, Kippenberg 2010, Warchalowski 2010), and is relatively well-known from chromosomal standpoints because 42 taxa have been surveyed to date and their range of diploid numbers goes from 2n = 18 to 2n = 44 (Gómez-Zurita et al. 2004, Petitpierre 2011).

Herein, we report the chromosome numbers, male sex-chromosome systems, and main features of their karyotypes of Timarcha (Metallotimarcha) metallica (Laicharting, 1781), Timarcha (Timarcha) carmelenae Petitpierre, 2013 and Timarcha (Timarcha) parvicollis ssp. seidlitzi Kraatz, 1879 to enlarge the cytogenetic analysis of the genus and discuss the most relevant trends of its chromosomal evolution.

## Material and methods

The three checked species and their geographical origins are given in Table 1. The chromosome analyses were only performed on male living individuals brought to our laboratory in Palma de Mallorca (Spain), where they were killed with ethyl acetate. The cytogenetic data were obtained by testis dissection of male adult specimens which were fixed in 45% acetic acid , later on teased into small pieces for five minutes, squashed under a coverslip, immediately frozen in liquid nitrogen to remove the coverslip, and finally treated using conventional Giemsa staining procedures. Most examined cells were at meiotic metaphase I, providing the male meioformulae, thus the number of autosomal bivalents plus the male sex-chromosome systems. Finally, we took micrographs by a ZEISS AXIOPHOT or a ZEISS AXIOSKOP photomicroscope, and subsequently enlarged them for printing.

**Table 1.** Chromosomally analysed species of *Timarcha* and their geographical sources. FR=France, SP=Spain.

*Timarcha metallica* (Laicharting, 1781)	Deville: Bois de Waibes, Ardennes (FR)
*Timarcha carmelenae* Petitpierre, 2013	P.N. Sierra de Castril: Sierra Seca, Granada (SP)
	‘’ La Sagra: collado de las Víboras, Granada (SP)
*Timarcha parvicollis seidlitzi* Kraatz, 1879	Sierra Tejeda: La Maroma, Granada (SP)

## Results

### Timarcha (Metallotimarcha) metallica (Laicharting, 1781)

**Figures 1–6.**
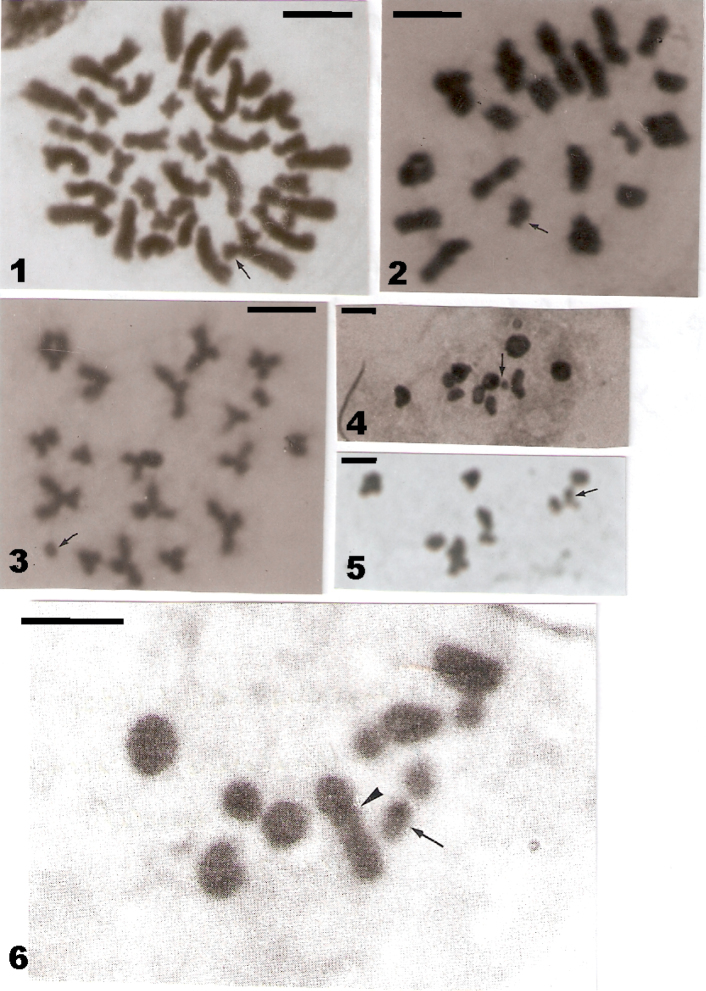
**1–3** *Timarcha metallica*: **1** spermatogonial mitotic metaphase with 2n = 38 chromosomes, the y-chromosome is arrowed **2** meiotic metaphase I with 18 + Xy_p_ meioformula, the Xy_p_ is arrowed **3** meiotic metaphase II with n = 19 chromosomes **4–5** *Timarcha carmelenae*: meiotic metaphases I from Sierra de Castril (**4**) and La Sagra (**5**) individuals, with 9 + Xy_p_ meioformula, the Xy_p_ are arrowed **6** Timarcha parvicollis ssp. seidlitzi: meiotic metaphase I with 11 + Xy_p_ meioformula, the Xy_p_ is arrowed and two partly overlapped autosomal bivalents are arrowheaded. Bar: 5 µm.

Two males of this species have displayed 2n= 38 chromosomes and an 18 + Xy_p_ male meioformula, with a “parachute” Xy_p_ sex-chromosome system (Fig. 2). Its karyotype is composed of nine medium size and nine small autosome pairs plus a submetacentric X-chromosome of medium size and a tiny y-chromosome. Four of the medium size autosome pairs were acrocentrics and the remaining meta- or submetacentrics, and three of the small ones were acrocentrics and the other metacentrics, as shown by spermatogonial mitotic metaphases (Fig. 1) and meiotic metaphases II (Fig. 3). Thus, the fundamental number (FN) of chromosomal arms is 50.

### Timarcha (Timarcha) carmelenae Petitpierre, 2013

One male individual from Sierra Seca and another from La Sagra provided meiotic metaphases I of 9 + Xy_p_, again with a “parachute” Xy_p_ sex-chromosome system, that is 2n = 20(Xy_p_) chromosomes, and showing two autosomal bivalents a bit larger than the others (Figs 4 and 5).

### Timarcha (Timarcha) parvicollis ssp. seidlitzi Kraatz, 1879

The only checked male individual provided meiotic metaphase I with an 11 + Xy_p_ meioformula, having also a “parachute” Xy_p_ sex-chromosome system, thus 2n = 24(Xy_p_), where five autosomal bivalents are larger than the remaining six ones (Fig. 6).

## Discussion

The diploid number of 2n = 38 chromosomes shown in Timarcha (Metallotimarcha) metallica should correct a previous miscounting report of 2n = 20 chromosomes (Petitpierre 1982). The high chromosome number found in this species is not displayed by any other *Timarcha* from the Palaearctic (subgenus *Timarcha* s.str.), whose range of numbers goes from 2n = 18 to 2n = 30 (Gómez-Zurita et al. 2004, Petitpierre 2011). However, high chromosome numbers are characteristic of the two species of the subgenus *Americanotimarcha* Jolivet, 1948, e.i., *Timarcha intricata* Halderman, 1854 with 2n = 44 (Petitpierre and Jolivet 1976) and *Timarcha cerdo* Stal, 1860 with 2n = 38 (Jolivet and Petitpierre 1992). These high chromosome numbers are in agreement with the similar morphological traits, the male genitalia and the molecular phylogenetic resemblances between the subgenera *Metallotimarcha* Motschulsky, 1860 and *Americanotimarcha* (Jolivet 1948, Iablokoff-Khnzorian 1966, Gómez-Zurita et al. 2000, Gómez-Zurita et al. 2004, Jolivet et al. 2013). Although the species of both subgenera show some plesiomorphic features, such an incomplete fusion of elytra, weak sexual dimorphism, aedeagus with a long tegmen cap, and a basal position in the molecular phylogenetic tree, their high chromosome numbers can not be considered as an ancestral character. First, because 2n = 20(Xy_p_) is assumed to be the plesiomorphic and most frequent karyotype condition for Coleoptera of the suborder Polyphaga (Smith and Virkki 1978, Angus et al. 2007). Besides, this is the most common karyotype in the genus *Timarcha* where more than a half of the 42 surveyed taxa show 2n = 20(Xy_p_) (Petitpierre 2011). And third, the karyotypes of both *Timarcha metallica* and *Timarcha intricata* share a quite high number of acrocentric autosome pairs, seven and fourteen respectively, which is an indication of their derived origin by multiple centric fissions or chromosomal dissociations from meta- or submetacentric chromosomes. Therefore, we assume that a hypothetic karyotype of 2n = 20(Xy_p_) chromosomes, mostly composed of metacentrics or submetacentrics, would have been the plesiomorphous state for the genus, from which all the taxa of the three present subgenera, *Americanotimarcha*, *Metallotimarcha* and *Timarcha* s.str. may have radiated.

The karyotype of Timarcha (Timarcha) carmelenae with 2n = 20(Xy_p_), with two larger autosomal bivalents and the remaining gradually decreasing, is similar to those of Timarcha (Timarcha) intermedia Herrich-Schäffer, 1838, and Timarcha (Timarcha) lugens Rosenhauer, 1856 (Petitpierre 1970, 1976). These three species share close morphological resemblances and a feeding on Brassicaceae plants, *Hormathophylla spinosa* (L.) Küpfer, 1974 for both Timarcha (Timarcha) carmelenae and Timarcha (Timarcha) lugens (González-Megías and Gómez 2001, Petitpierre and Daccordi 2013) and *Carrichtera annua* (L.) DeCandolle, 1821 for Timarcha (Timarcha) intermedia (Petitpierre 1971, Jolivet and Petitpierre 1973), in contrast with the prevalent trophism on plants of Rubiaceae and/or Plantaginaceae reported for almost all the other taxa of the subgenus *Timarcha* s.str. (Jolivet and Petitpierre 1973).

Timarcha (Timarcha) parvicollis ssp. seidlitzi shows a karyotype of 11 + Xy_p_ male meioformula, thus 2n = 24(Xy_p_) chromosomes, which separates it strikingly from the related Andalusian species with 2n = 20(Xy_p_) such as Timarcha (Timarcha) insparsa Rosenhauer, 1856, Timarcha (Timarcha) marginicollis Rosenhauer, 1856, Timarcha (Timarcha) intermedia, Timarcha (Timarcha) lugens Rosenhauer, 1856 and Timarcha (Timarcha) carmelenae, sharing a bifid mesosternum and elytra covered with spare and fine puncturation.

Another species of *Timarcha* with 2n = 24 chromosomes, Timarcha (Timarcha) pratensis (Duftschmid, 1825) (Petitpierre 1976), from Central and Eastern Europe, and Northern Italy, belongs to a very different group without any close interrelationship with Timarcha (Timarcha) parvicollis (Bechyné 1948, Warchalowski 2003).
